# Estradiol Regulates Txnip and Prevents Intermittent Hypoxia-Induced Vascular Injury

**DOI:** 10.1038/s41598-017-10442-7

**Published:** 2017-09-04

**Authors:** Xiao Fei Lan, Xiu Juan Zhang, Ying Ni Lin, Qiong Wang, Hua Jun Xu, Li Na Zhou, Pei Li Chen, Qing Yun Li

**Affiliations:** 1 Department of Respiratory Medicine, Ruijin Hospital, Shanghai Jiao Tong University School of Medicine, No. 197 Ruijin Er Road, Shanghai, 200025 China; 2 Department of Respiratory Medicine, Shanghai Tongren Hospital, Shanghai Jiao Tong University School of Medicine, No. 1111 West Xianxia Road, Shanghai, 200335 China; 3 Department of Respiratory Medicine, Huashan Hospital, Fudan University School of Medicine, No.12 Middle, Urumqi Road, Shanghai, 200040 China; 4 Department of Otolaryngology, Shanghai Jiao Tong University Affiliated Sixth People’s Hospital, Otolaryngology Institute of Shanghai Jiao Tong University, No. 600 Yishan Road, Shanghai, 200233 China

## Abstract

Chronic intermittent hypoxia (IH) contributes to obstructive sleep apnea (OSA)-related cardiovascular diseases through increasing oxidative stress. It has been widely recognized that estradiol decreases the risk for cardiovascular disease, but the estrogen replacement therapy is limited for its side effects. Thioredoxin (Trx) and its endogenous inhibitor, thioredoxin-interacting protein (Txnip), are associated with the protective effect of estradiol in some conditions. In this study, we aimed to explore whether estradiol could protect against IH-induced vascular injury, and the possible effect of Trx-1/Txnip in this process. Forty-eight adult female C57/BL6J mice were randomly divided into 4 groups, ovariectomy combined with IH group, sham operation combined with IH group, IH group and the control group. The mice treated with IH for 8 hrs/day, and 28 days. IH induced the injury of aorta, and ovariectomized mice were more prone to the IH-induced aortic injury, with higher level of oxidative stress. *In vitro*, estradiol increased Trx-1 level, but decreased the level of Txnip and oxidative stress in human umbilical vein endothelial cells (HUVECs) treated with IH for 16 hrs. Knock-down of Txnip by specific siRNA rescued oxidative stress and apoptosis. In conclusion, estradiol protects against IH-induced vascular injury, partially through the regulation of Trx-1/Txnip pathway.

## Introduction

Characterized by repetitive episodes of upper airway collapse during sleep, obstructive sleep apnea (OSA) affects about 4% of men and 2% of women^1^. Chronic intermittent hypoxia (IH) caused by repetitive upper airway collapse contributes to systemic complications including cardiovascular diseases (CVD)^2, 3^. As the first-choice treatment of OSA, continuous positive airway pressure (CPAP) decreases the risk for CVD^4^. However, poor compliance with CPAP therapy is quite a common clinical problem. Thus, the novel prevention and treatment strategies for CVD in OSA patients should be concerned. Series of clinical evidences support the protective effect of estrogen against CVD^5, 6^. A prospective survey on 121,964 female nurses favored the postmenopausal estrogen use to reduce the risk for severe CVD^5^. However, long-term estrogen replacement therapy increases the risk for endometrial cancer and breast cancer^6^. Therefore, uncovering the underlying mechanisms of cardiovascular protection of estrogen will be helpful for the development of new therapeutic targets for the CVD complication in OSA patients.

The increased oxidative stress is the main mechanism of OSA associated CVD. The oxidative stress injury is caused by the imbalance between oxidation and reduction system, with excessive reactive oxygen species (ROS). Trx and Txnip play an essential role in the regulation of oxidative stress level. Trx-1 mainly exists in cytoplasm, with anti-oxidative effect mediated by the dithiol-disulfide exchange in the active site -Cys-Gly-Pro-Cys-. As an endogenous inhibitor of Trx, Txnip has been found to modulate the oxidative stress through binding to the active center of Trx.

Previous study indicated that estrogen protects against CVD through preventing oxidative stress-associated endothelial cell apoptosis^7^. It was also shown that the protective effect of estrogen was associated with the Trx-1 and Txnip^8, 9^. Thus, it’s reasonable to infer that estrogen could protect against CVD through Trx-1/Txnip pathway in OSA patients. Herein, we established the IH mouse and cell models to explore the role of estrogen on IH-induced vascular injury, and its association with Trx-1/Txnip.

## Results

### Ovariectomy promoted the IH-induced vascular injury

**Figure 1:**
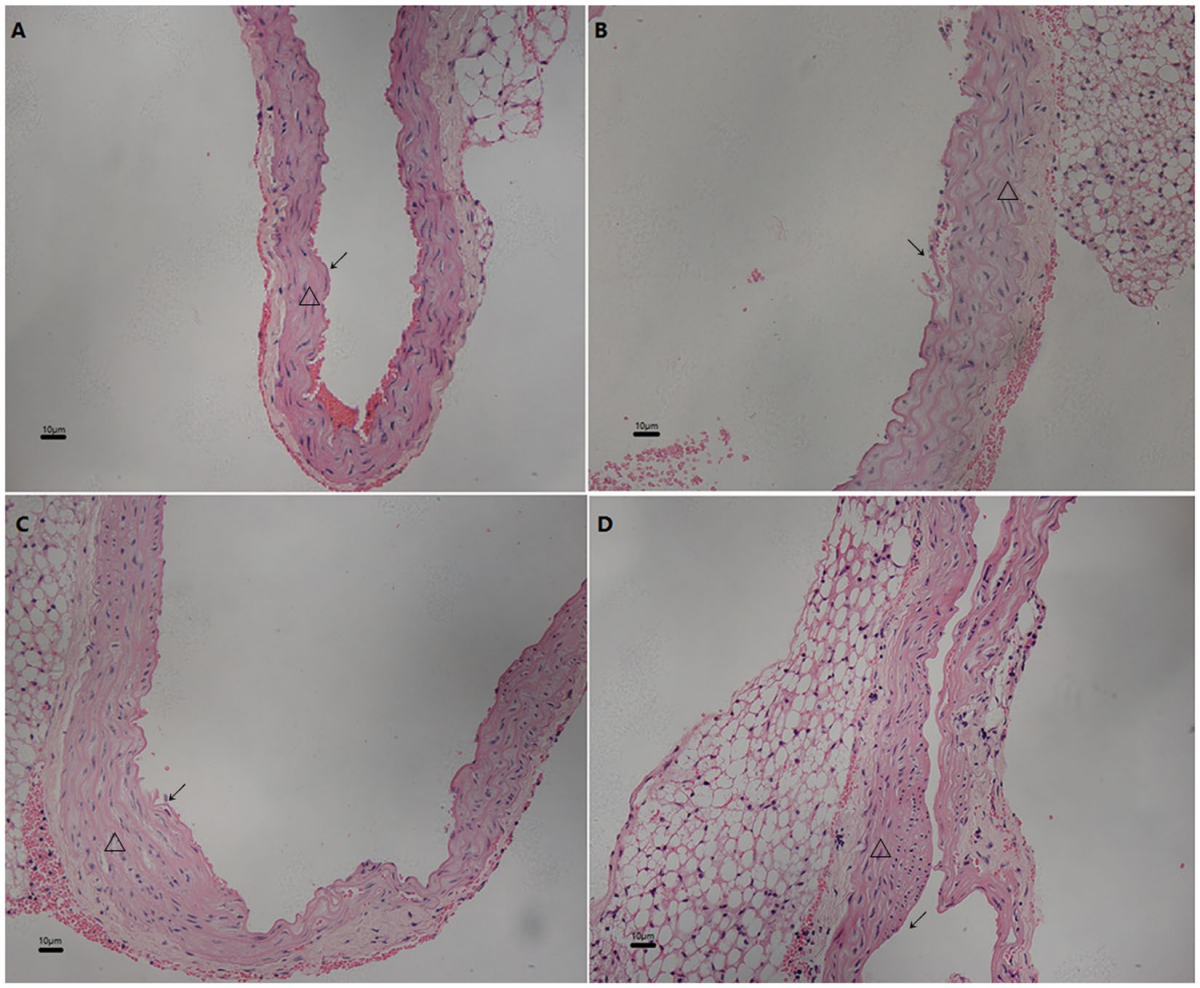
Ovariectomy promoted the IH-induced aortic injury (HE 100×). The morphology of aorta arch by HE staining presented as derangement, enlargement and proliferation of smooth muscle cells and the disrupted endothelium in mice treated with IH alone (**B** and **C**) or combined with the estradiol deficiency after ovariectomy (**D**). The early sign of atherosclerosis, the thickening of intima, was observed in mice with the combined treatment of IH and estradiol deficiency (**D**). The morphology of aorta in control group was normal (**A**). IH: intermittent hypoxia. ↙ Endothelial cell; Δ Vascular smooth muscle.

The ovariectomy was conducted to induce the deficiency of estrogen in female adult mice. The derangement, enlargement and proliferation of smooth muscle cells, and the disrupted aortic endothelium were observed in mice treated with IH alone (Fig. 1B and C) or combined with ovariectomy (Fig. 1D). Additionally, the thickening of intima, an early sign of atherosclerosis, presented in mice treated with both chronic IH and ovariectomy (Fig. 1D).

### Ovariectomy increased the intima-media thickness (IMT)

**Figure 2:**
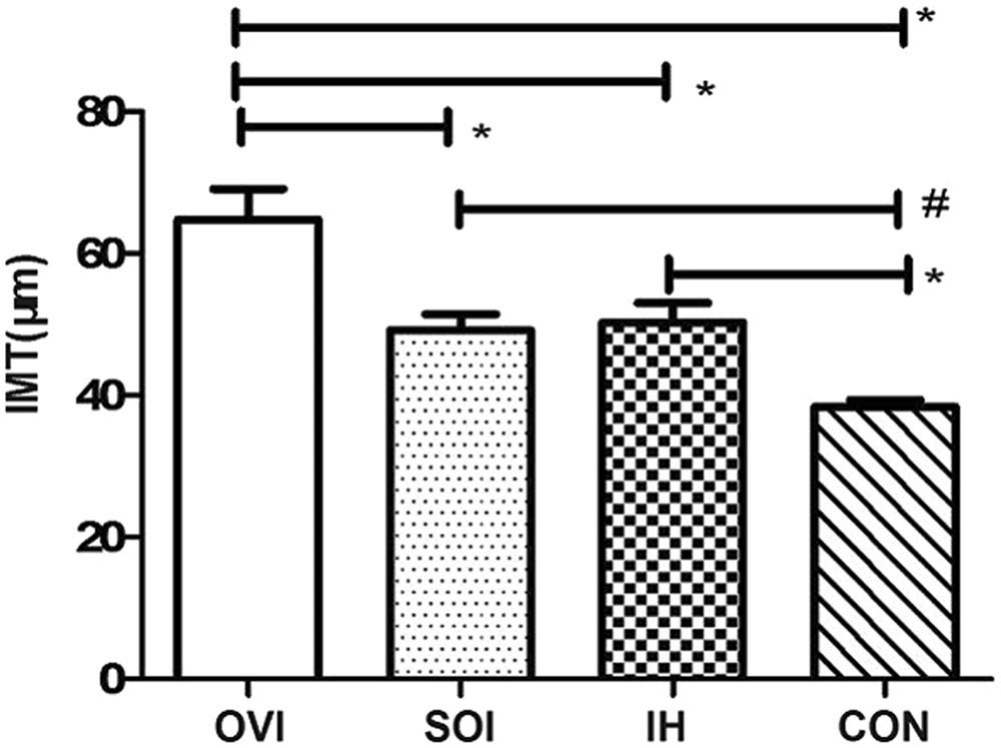
Ovariectomy increased the IMT of aorta in mice under IH. The chronic IH treatment (IH group) increased the IMT of aorta than that in control group (n = 6, *P < 0.01). In addition, the IH and ovariectomy treatments (OVI group) further significantly increased the IMT, in contrast to the mice treated with IH and sham-operation (n = 6, *P < 0.01). IMT: intima-medial thickness. IH: intermittent hypoxia.

IMT is a subclinical biomarker of CVD. We found that the chronic IH treatment increased the IMT of aorta, when compared with the control. The combined treatment of ovariectomy and IH presented increased IMT than IH treatment alone (Fig. 2). These results indicated that estradiol efficiency caused by ovariectomy promoted IH-induced vascular injury.

### Ovariectomy increased the level of plasma malondialdehyde (MDA)

**Figure 3:**
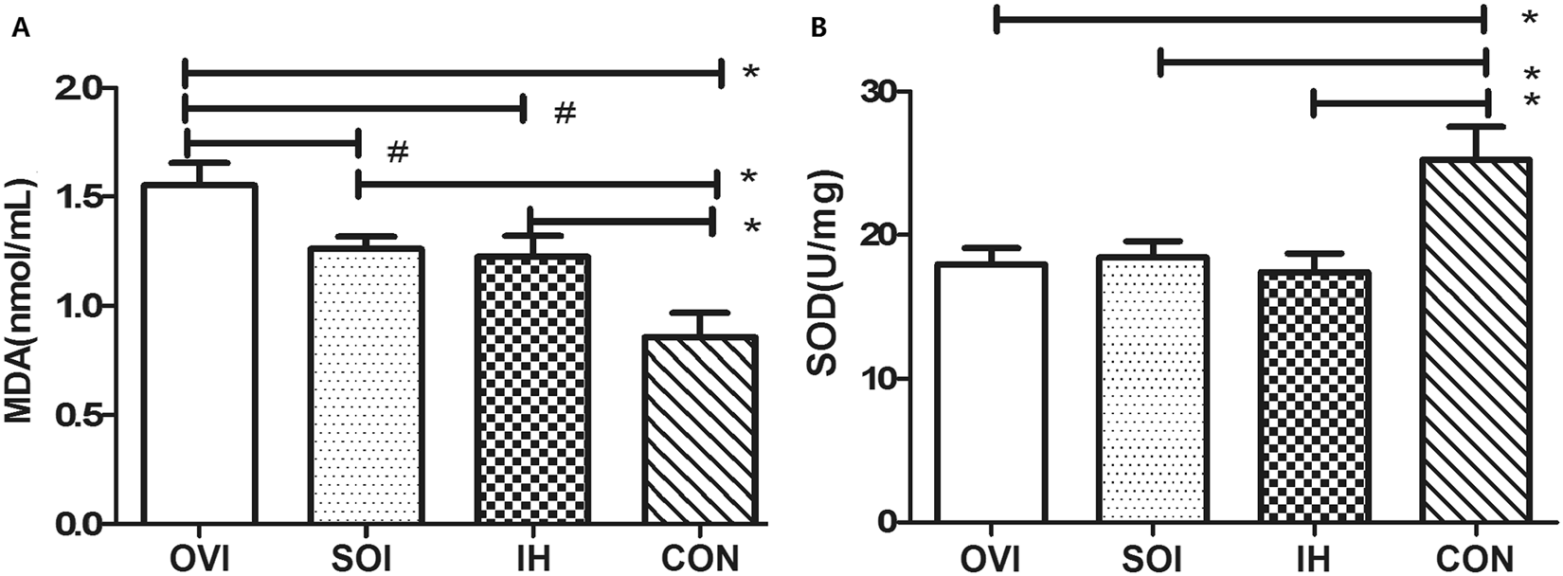
Ovariectomy increased the MDA level under IH model. Compared with control (CON group), higher levels of MDA in plasma and lower level of SOD activity in aorta tissue were induced by IH (IH group) in mice (n = 12, *P < 0.01, **A** and **B**, respectively). Besides, the MDA level increased more significantly in mice treated with IH and ovariectomy than in that only treated with IH (n = 12, *P < 0.01, **A**). MDA: malondialdehyde; SOD: superoxide dismutase.

MDA is generally considered as a biomarker of oxidative stress level, and Superoxide Dismutase (SOD) is one of the most important anti-oxidation substances. We found higher level of MDA in plasma of mice treated with IH, and much more significant in IH mice further treated with ovariectomy (Fig. 3A). Besides, the total SOD activity was significantly lower in mice under chronic IH than the control. No further difference was found in SOD activity caused by ovariectomy (Fig. 3B).

### Estradiol rescued cell apoptosis and oxidative stress ***in vitro***

**Figure 4:**
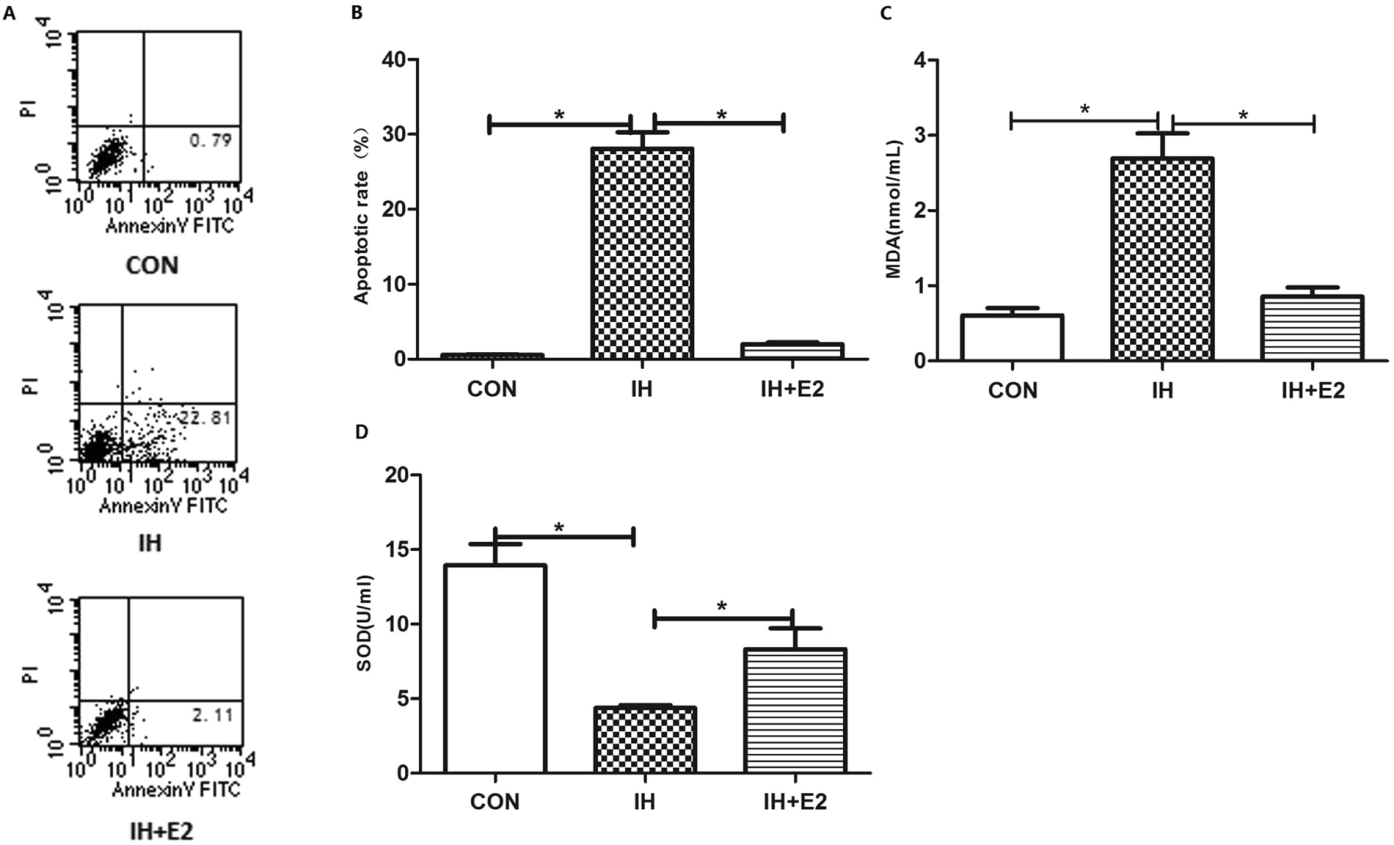
Estradiol reduced the IH-induced cell apoptosis and oxidative stress *in vitro*. IH significantly increased the apoptotic rate of HVUECs and the MDA level in supernatants, which were completely rescued by estradiol supplement (n = 5, *P < 0.01, **A** and **B**, respectively). (**C**) Besides, estradiol significantly increased the SOD activity which is reduced by the IH treatment (n = 5, *P < 0.01). MDA: malondialdehyde; SOD: superoxide dismutase. E_2_: estradiol.

After 16 hrs of IH exposure, the apoptotic rate of HUVECs increased significantly (Fig. 4A). The apoptosis of HUVECs under IH was completely reduced by estradiol (Fig. 4A). The MDA level in supernatants of HUVECs increased significantly, while the activity of total SOD decreased after IH treatment for 16hrs (Fig. 4B). Estradiol partially rescued the IH-associated increased oxidative stress, as supported by the decreased MDA level and increased activity of SOD (Fig. 4C).

### Estradiol increased Trx-1 and decreased Txnip expressions *in vitro*

**Figure 5:**
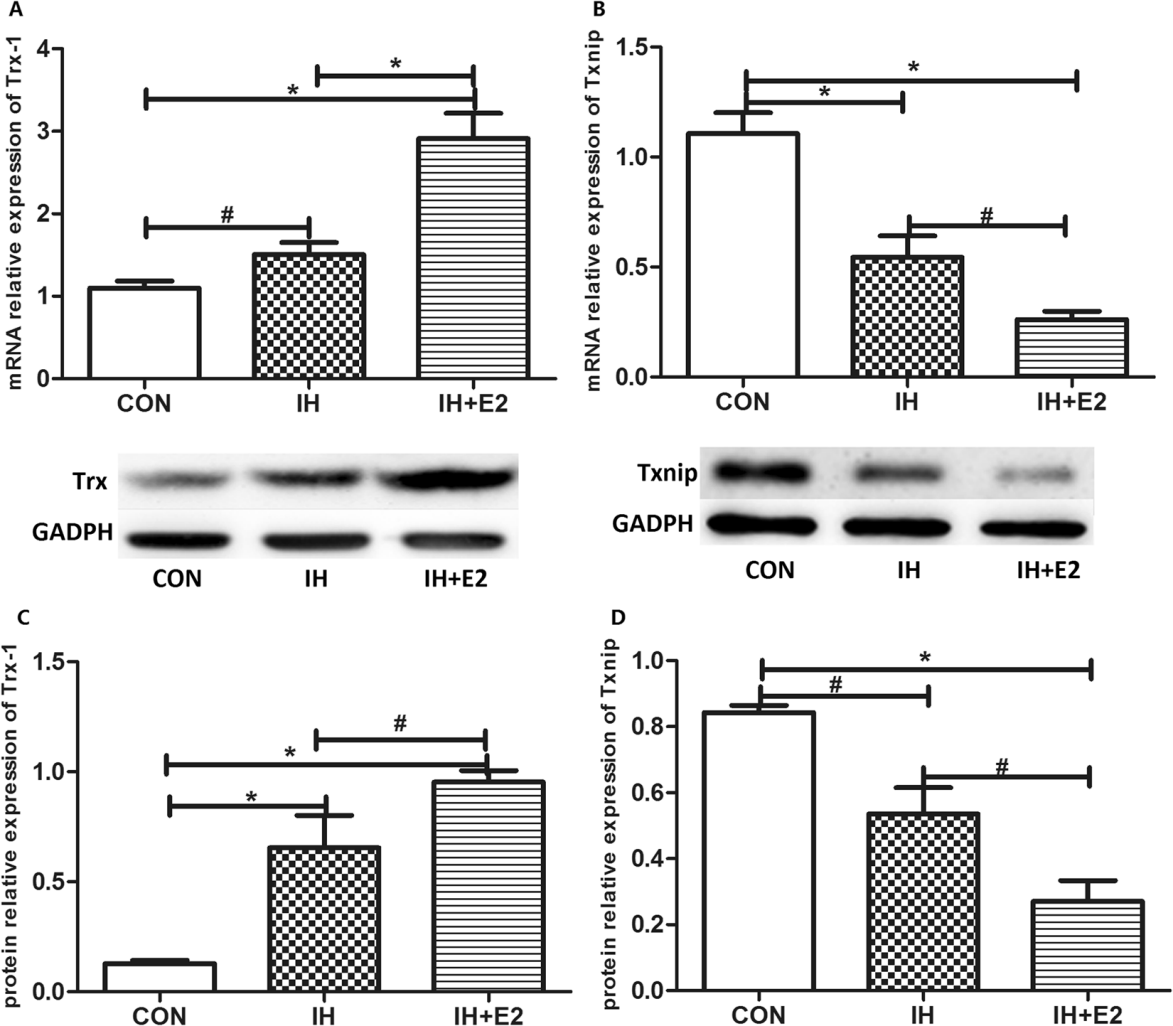
Estradiol increased Trx-1 and reduced Txnip expressions. (**A**) The mRNA and protein levels of Trx-1 increased significantly in HUVECs after 16hrs of IH, which were further elevated by estradiol supplement (n = 5, ^#^ *P* < 0.05 and *P < 0.01). (**B**) IH treatment inhibited the mRNA and protein expressions of Txnip in HUVECs, which were more significant combined with estradiol treatment (n = 5, ^#^ *P* < 0.05 and *P < 0.01).

The IH induced the expression of Trx-1 mRNA and protein levels, and estradiol further elevated the expression of Trx-1 (Fig. 5A and C). The mRNA and protein levels of Txnip were down-regulated by IH, and estradiol further decreased Txnip expression (Fig. 5B and D).

### Knock-down of Txnip decreased IH-induced cell apoptosis and oxidative stress

**Figure 6:**
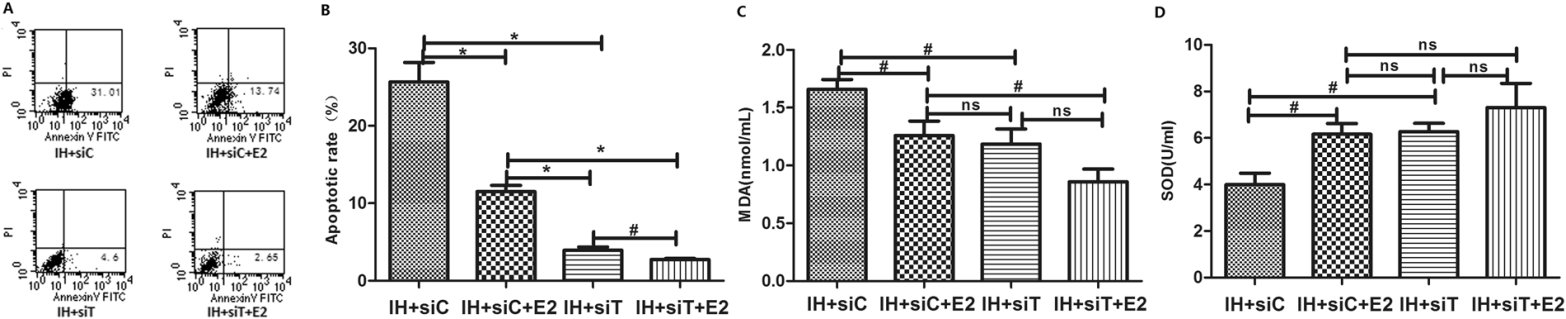
Knock-down of Txnip suppressed the apoptosis of HUVECs more significant than E_2_, while had no difference in oxidative stress. Knock-down of Txnip significantly suppressed the IH-induced apoptosis of HUVECs, and further reduced apoptosis combined with estradiol treatment (n = 5, ^#^ *P* < 0.05 and *P < 0.01, (**A** and **B**). The level of MDA decreased (**B**) and the SOD activity (**C**) increased when treated with knock-down of Txnip or estradiol (n = 5, ^#^ *P* < 0.05). While, no difference was found in the levels of MDA and SOD between knock-down of Txnip and estradiol treatment (n = 5, ns *P* > 0.05, (**C** and **D**), implying effects of estradiol partially through inhibition of Txnip. siT: siTxnip; siC: cont-siRNA.

In order to investigate the effect of Txnip on IH-induced vascular injury, we knocked down Txnip by specific siRNA. It was found that apoptotic rate decreased significantly when treated with Txnip knockdown or estradiol alone and further decreased under treatment of Txnip knockdown and estradiol (Fig. 6A and B). Knock-down of Txnip reduced MDA level and elevated SOD activity, similar to effects of estradiol (Fig. 6C and D). However, the level of MDA and activity of SOD showed no further reduction under treatment of Txnip knockdown combined with estradiol.

## Discussion

OSA is considered as an independent risk factor for CVD^10^. Our study found that the IH, the main characteristic of OSA, increased IMT and contributed to the injury of endothelial cells and proliferation of vessel smooth muscle cells. *In vitro*, we further confirmed that estradiol treatment reduced the apoptosis of HUVECs.

To explore the effect of estradiol on vascular injury, the ovariectomy was performed to induce the deficiency of estrogen in adult female mice, as reported in previous study^11^. The increased IMT and vascular injury, regarded as subclinical markers for atherosclerosis, are positive correlated with the increased risk for OSA-related CVD^12–15^. These results suggested that estradiol prevented the IH-induced vascular injury, which was consistent with the previous studies^16, 17^. Estrogen protects against vascular injury and atherosclerosis mainly through directly binding to estrogen receptor-alpha (ERα). In human research, increased level of ERα in the vasculature of pre-menopausal women correlated with low atherosclerosis incidence rates^18^. The protective role of estrogen against vascular injury diminished after knocking down of ERα in ovariectomized female mice^19^. In addition, membrane-bound G protein-coupled estrogen receptor (GPER-1) is found to be a regulator of endothelial inflammation, which could also be a potential therapeutic target against atherosclerosis^20^. Although the effects of estrogen on ovariectomized female mice or the male mice have not been further explored in our study, the cardiovascular effect of estradiol are both seen in male and ovariectomized female animals in previous study^21^. The estradiol was also found in human to regulate the vascular function in male^22^. This may be explained by the widely existence of ERs in vascular cells both in female and male.

Further, our results also showed increased MDA level and decreased SOD activity under IH treatment. Besides, estradiol deficiency increased MDA level, which was rescued by estradiol *in vitro*. MDA is one of the most important products of lipid peroxides, further oxidizing low density lipoprotein (LDL), enhancing the atherosclerosis. Estradiol has been reported to prevent the oxidation of LDL, the disruption of the endothelial barrier initiated by vascular accumulation of modified LDL^23^ and inhibit macrophage uptake of acetylated LDL^24^. Although the molecular mechanism of vascular responses to estradiol has not been established well, some evidences show that estradiol protects against vascular injury through reducing oxidative stress^25, 26^. SOD is an enzyme catalyzing superoxide anion into H_2_O_2_ and eliminates the ROS. Estradiol has been found to inhibit NADPH oxidase expression^27^ and stimulate the expressions of the anti-oxidation substances, such as manganese SOD^28^ to reduce oxidative stress. It was reported that SOD activity of heart homogenates decreased over time in rats treated with IH^29^, which is consistent with our finding. Thus, the increased oxidative stress level might be associated with the IH-induced vascular injury, and estradiol is effective in preventing this process.

As a key determinant of cellular sulfhydryl redox homeostasis, Trx has been found to be involved in a variety of diseases. Higher level of Trx-1 was observed in patients with high risk factors for CVD^30^. In our study, higher level of Trx-1 expression was induced by IH, consistent with previous finding of increased Trx level in OSA patients^31^. As a negative regulator of Trx, Txnip increased the susceptibility of cell to oxidative stress, resulting in higher rate of cell apoptosis^32, 33^. The inhibition of Txnip may prevent from cardiac injury, through promoting the antioxidant and anti-apoptosis effects of Trx-1^34^. It was found lower level of Trx and higher level of Txnip in male than in female mice under IH^35^. In addition, estradiol acted as an antioxidant substance by increasing the expression of Trx and decreasing the expression of Txnip in the uterus of mice^9^. Yuan *et al*. also found the increased expression level of Trx in ovariectomized female mice treated with estradiol^36^. Thus, the increased Trx-1 and decreased Txnip levels might be regulated by estradiol to prevent IH-induced vascular injury.

During the past decade, Txnip has been studied as a potential pharmacotherapeutic target in cardiovascular disease^37^. In this study, Txnip was knocked down in HUVECs to explore the effect of Txnip on oxidative stress and apoptosis. The results indicated lower MDA level and higher total SOD activity level were achieved by knocking down of Txnip, similar to effects of estradiol treatment. This result suggested estradiol could decrease the oxidative stress through reducing the expression of Txnip. Txnip has been found to induce apoptosis via disrupting the Trx-ASK1 complex, which further actives the ASK1-c-jun-N-terminal kinase (JNK) and p38MAP kinase pathway^38^. Besides, it also induces the release of pro-inflammatory mediators such as TNF-α or IL-1β, and results in cell cycle arrest^37^. In view of this, Txnip might be a key regulator during IH-induced vascular injury, and it needs to be further studied.

Limitations should be mentioned in this study. First of all, these findings are lack of studies in male or in ovariectomized female mice treated with estradiol. Secondly, analysis of subgroups of SOD is essential to distinguish the source from mitochondria or cytoplasm. Finally, estradiol receptor mechanisms involved in the regulation of Trx-1/Txnip and oxidative stress need to be explored further.

In summary, the decreased oxidative stress level and apoptosis rate are associated with protection effect of estradiol on vascular injury induced by IH, involving the regulation of Trx-1 and Txnip in this process. Additionally, knock-down of Txnip significantly reduced the cell apoptosis rate, which is partially associated with estradiol. Thus, our study implies that estradiol protects against IH-induced vascular injury, partially through the regulation of Trx-1/Txnip pathway.

## Material and Methods

### Animal model

The animal study was approved by the Committee for Research and Ethics of Ruijin Hospital, affiliated to Shanghai Jiao Tong University School of Medicine in accordance with NIH guidelines. A total of 48 adult female C57/BL6J mice (aged 6–8 weeks, obtained from Lake Hayes Laboratory Animal Co., Ltd. Shanghai, China) were randomly divided into 4 groups: the ovariectomized IH group (OVI group), the sham-operation IH group (SOI group), IH group and the control group (CON group). Mice in the former 3 groups were exposed to IH (5% O_2_ for 10 s followed by 21% O_2_, 40 cycles/h) and mice in CON group were exposed to intermittent air. These mice were exposed to IH or air for 8 hrs/d, and 28 days. Mice in OVI group underwent bilateral ovariectomy under 1.5% isoflurane inhalational anesthesia as described previously^39^. The SOI group underwent the sham-operation. At day of 29, all mice were euthanatized by cervical dislocation^40^.

### Hematoxylin-eosin staining and IMT measurement of aorta arch

Hematoxylin-eosin (HE) staining of aorta arch tissue followed the methods as described before^41^. IMT of aorta arch was measured by the image analysis software (Image-Pro Plus6.0, Media Cybernetics, USA). The IMT was measured in two vascular cross sections in 6 samples of each group.

### Measurements of SOD activity and MDA

The oxidative stress associated biomarkers include MDA and the activity of SOD. SOD activities in the arterial tissues and in the supernatants of HUVECs, and the levels of MDA in plasma of mice and in the supernatants of HUVECs were measured using SOD assay kit and MDA assay kit (Nanjing Jiancheng Technology Co., Ltd., Nanjing, China) according to the manufacturer’s protocols.

### IH exposure and 17β-estradiol treatment in HUVECs

HUVECs (ECV304) (obtained from Blood Research Institute, Shanghai, China) were cultured in DMEM medium supplied with 10% fetal bovine serum (FBS, Gibco, USA) in a humidified incubator under 5% CO_2_ at 37 °C. The cells were divided into the following three groups: IH group (cells were exposed to IH), IH + E_2_ group [cells were exposed to IH with the treatment of 10^−7^mol/L 17β-estradiol (Sigma, USA)], and control (CON) group. IH exposure (1% O_2_ for 5 min followed by 21% O_2_ for 5 min, 6 cycles/h, and 16 hrs) was controlled by a self-designed computer program, while CON group was treated with intermittent air. Cells used *in vitro* experiments were from the same batch, and the results were repeated for five times.

### Measurement of cell apoptosis

HUVECs were digested by 0.25% trypsin after removing the supernatants. Apoptosis rate was tested by flow cytometer (BD, USA) using Annexin V-FITC Apoptosis Detection Kit (BD, USA) according to manufacturer’s protocol.

### RNA extraction and quantitative real-time PCR

Total RNAs were extracted from HUVECs using trizol (Gibco, USA). cDNAs were synthesized using a reverse transcriptional system. Quantitative real-time PCR (qRT-PCR) was performed using SYBR (Takara Bio, Dalian, China) according to the manufacturer’s instructions. The values were normalized to the level of β-actin. The primers used in qRT-PCR were listed as follows:

Trx-1 F: 5′- CAA GAT GGT GAA GCA GAT-3′

Trx-1 R: 5′- GTG GCT GAG AAG TCA ACT A-3′

Txnip F: 5′- CCG AGC CAG CCA ACT CAA G-3′

Txnip R: 5′- ACA CCC GCC CAT CAG GAA T-3′

β-actin F: 5′- AAG GTG ACA GCA GTC GGT T-3′

β-actin R: 5′-TGT GTG GAC TTG GGA GAG G-3′

### Western blotting

HUVECs were washed with PBS, harvested by lysis buffer (Beyotime, China), and then centrifuged at 1500 × *g* at 4 °C for 15 mins. The concentration of total proteins was measured by bicinchoninic acid (BCA) kit (Thermo Scientific, USA). Samples (containing 30 µg proteins) were boiled in 2 × loading buffer, separated by a 12% sulfate-polyacrylamide gel (PAGE-SDS), and transferred to a polyvinylpyrrolidone difluoride (PVDF) membrane. The membranes were incubated with antibodies for Trx-1 (Trx-1 rabbit anti-human polyclonal antibody, 1:1,000 dilution; Sigma, USA), Txnip (Txnip mouse anti-human polyclonal antibody,1:500 dilution; Novus, USA), and GADPH (GADPH rabbit anti-human polyclonal antibody,1:1,000 dilution; Sigma, USA) overnight at 4 °C after blocking with phosphate saline buffer containing 0.1% (v/v) Tween and 5% (w/v) dried milk. Then the membranes were washed for 3 times and incubated with second antibodies (goat anti-rabbit IgG, 1:1,000 dilution; Sigma, USA; goat anti-mouse IgG, 1:1,000 dilution; Sigma, USA; respectively) for 1 hr at room temperature. After washing for 3 times, membranes were incubated with chemiluminescence (ECL, Thermo Scientific, USA) for 1 min before exposure to the medical X-ray films.

### Transfection with siRNAs

HUVECs were seeded in 6-well plates 24 hrs before transfection. Control small interfering RNA (cont-siRNA) or specific siRNA against Txnip (siTxnip) were transfected by Lipofectamine 2000 (Invitrogen, UK). The sequence of cont-siRNA was 5′-UUC UCC GAA CGU GUC ACG UTT-3′, and the sequence of siTxnip was 5′-GCC CUU AGG AUC CUG GCU UTT-3′. Forty-eight hours after transfection, cells were exposed to IH (IH + cont-siRNA group and IH + siTxnip group), or IH with the treatment of 17β-estradiol (IH + cont-siRNA + E_2_ group and IH + siTxnip + E_2_ group). The apoptosis rate and oxidative stress level were detected as mentioned before.

### Statistical Analysis

SPSS 20.0 (SPSS, Chicago, IL, USA) software was used for data analysis. Measurement data was showed as Mean ± SEM. For the normally distributed data, one-way ANOVA was used for comparison among multiple groups and LSD post hoc test was performed for comparison between two groups. For the data that were not normal distribution, Kruskal-Wallis H rank sum test was performed for the comparisons among multiple groups. *P* value < 0.05 was considered as statistically significant.
